# Donor-derived IL-17A and IL-17F deficiency triggers Th1 allo-responses and increases gut leakage during acute GVHD

**DOI:** 10.1371/journal.pone.0231222

**Published:** 2020-04-06

**Authors:** Ivan Odak, Alina Depkat-Jakob, Maleen Beck, Michael Jarek, Yan Yu, Ursula Seidler, Sascha David, Arnold Ganser, Reinhold Förster, Immo Prinz, Christian Koenecke

**Affiliations:** 1 Institute of Immunology, Hannover Medical School, Hannover, Germany; 2 Department of Hematology, Hemostasis, Oncology and Stem-Cell Transplantation, Hannover Medical School, Hannover, Germany; 3 Helmholtz Center for Infection Research, Braunschweig, Germany; 4 Department of Gastroenterology, Hepatology and Endocrinology, Hannover Medical School, Hannover, Germany; 5 Department of Nephrology, Hannover Medical School, Hannover, Germany; Universite Paris-Sud, FRANCE

## Abstract

IL-17A and IL-17F cytokines are important regulators of acute graft-versus-host-disease (GVHD). However, contrary effects of these cytokines in inflammatory diseases have been reported. To investigate the effects of donor-derived IL-17A and IL-17F on GVHD, we made use of single (*Il17a*^-/-^ or *Il17f*^-/-^) and double deficient (*Il17af*^-/-^) allogeneic donor CD4^+^ T cells. We could demonstrate that transplantation of *Il17af*^-/-^ CD4^+^ donor T cells led to aggravated GVHD. However, this phenotype was not observed after transplantation of single, *Il17a*^-/-^ or *Il17f*^-/-^, deficient CD4^+^ T cells, suggesting redundant effects of IL-17A and IL-17F. Moreover, *Il17af*^-/-^ cell recipients showed an increase of systemic IFNγ, indicating a heightened pro-inflammatory state, as well as infiltration of IFNγ-secreting CD4^+^ T cells in the recipients’ intestinal tract. These recipients exhibited significant gut leakage, and markedly macrophage infiltration in the gastrointestinal epithelial layer. Moreover, we saw evidence of impaired recovery of gut epithelial cells in recipients of *Il17af*^*-/-*^ CD4^+^ T cells. In this study, we show that IL-17A/F double deficiency of donor CD4^+^ T cells leads to accelerated GVHD and therefore highlight the importance of these cytokines. Together, IL-17 cytokines might serve as a brake to an intensified Th1 response, leading to the exacerbated gut damage in acute GVHD.

## Introduction

Acute Graft-versus-Host disease (GVHD) is still a major cause of non-relapse-related mortality after allogeneic hematopoietic stem-cell or bone marrow transplantation (BMT) [1]. The current standard of care for higher grade GVHD is the systemic use of steroids. Further therapeutic options, especially for steroid-refractory GVHD are sparse [2]. Therefore, identification of new therapeutic targets both for prophylaxis and treatment of GVHD are needed. Allogeneic donor lymphocytes induce and orchestrate this highly inflammatory disease in the lympho-hematopoietic compartment and in GVHD target organs, respectively. In particular CD4^+^ T cells show a high degree of plasticity in the course of the disease [3]. Functional roles for Th1, Th2 and regulatory T cells (Tregs) are well known in GVHD [4,5]. However, the exact role of IL-17 and Th17 cell responses in acute GVHD is less clear.

The subset of CD4^+^ T cells termed Th17 cells is characterized by production of its signature cytokine IL-17A. However, the IL-17 cytokine family comprises IL-17A, IL-17B, IL-17C, IL-17D, IL-17E and IL-17F, all having a similar protein structure and sharing between 62% to 88% of homology of murine to human [6]. The corresponding IL-17 receptor family consists of five members, IL-17RA, IL-17RB, IL-17RC, IL-17RD and IL-17RE. IL-17RA forms a heterodimer with IL-17RC, which together binds IL-17A dimers, IL-17F dimers, as well as IL-17A:IL17F heterodimers [7,8]. IL-17A and IL-17F share 55% homology on the amino acid level, and are syntenic both in mice and humans [9]. Both cytokines are involved in anti-fungal, bacterial and allergic immune responses [10,11]. However, despite the apparent similarities, there is evidence for distinct roles of the two cytokines in immunity [12]. Depending on the experimental model, IL-17 cytokines IL-17A and IL-17F may exert either pathogenic or protective effects, e.g. promoting respiratory allergy [11] or mediating protection in nephritis [13]. To date, Janus-head roles taken by Th17 and associated cytokines such as IL-17A and IL-22 during acute GVHD have been documented [14] In one study, IL-17A deficiency led to disease reduction [15], whereas another study showed that the absence of IL-17A- secreting cells exacerbated GVHD [16]. However, experimental setups and GVHD models differed in those studies. IL-17A is proposed to exert a protective role during gut-inflammation by limiting excessive permeability and thereby maintaining barrier integrity [17,18]. Another protective role in colitis model has been attributed to IL-17A by forcing the expression of Th1- associated responses [19]. Since excessive endothelial and epithelial permeability is one of the prerequisites for acute GVHD [20], we hypothesized that donor-derived IL-17 cytokines exert a protective role in acute GVHD.

In this study, we dissect the role of donor-derived IL-17A and IL-17F for endothelial and epithelial permeability in an experimental acute GVHD model using single- (*Il17a*^*-/-*^, *Il17f*^*-/-*^) and double-deficient (*Il17af*^*-/-*^) donor T cells. Our results show a protective role of mutually redundant donor-derived IL-17 cytokines IL-17A and IL-17F. We further demonstrate that increased gut leakage and macrophage infiltration occurs when donor-derived IL-17A and IL-17F are absent. Our results suggest that donor-derived IL-17 might contribute to protection of the intestinal barrier during acute GVHD.

## Material and methods

### Animals

Wildtype (WT) C57BL/6 Thy1.2 (BL6, H-2K^b^), BALB/c (H-2K^d^), B6xDBA2 F1 (BDF1, H-2K^bxd^) mice were obtained from Charles River Laboratories (Sulzfeld, Germany). B6.129P2-Il17a^tm1Yiw^ (*Il17a*^*–/–*^) mice were kindly provided by Y. Iwakura (The University of Tokyo, Bunkyō-ku, Japan) B6.129S6-Il17f^tm1Awai^ (*Il17f*^*–/–*^) mice were kindly provided by B. Becher (University of Zürich, Zürich, Switzerland). WT C57BL/6 Thy1.1, *Il17a*^*–/–*^, *Il17f*^*–/–*^and *Il7af*^*-/-*^ (C57BL/6J-Il17a/Il17ftm1Impr) were bred at the central animal facility of Hannover Medical School under specific pathogen-free conditions. All animal experiments were carried out in accordance with institutional and governmental directives and were approved by Niedersächsisches Landesamt für Verbraucherschutz und Lebensmittelsicherheit (permit number: 33.14-42502-04-11/0619 and 33.19-42502-04-14/1660).

### Bone marrow transplantation and GVHD induction

For BMT and GVHD-induction in the C57BL/6→BALB/c model, 8–10 weeks old BALB/c recipients received lethal irradiation with 8 Gy from a Cs γ-source. Donor cells were transplanted within 24 hours after irradiation. All recipient mice received 3.0–5.0x10^6^ T cell-depleted bone marrow (TCD BM) C57BL/6 or BALB/c BM cells and 0.5x10^6^ CD4^+^ T cells from C57BL/6 WT, *Il17a*^*–/–*^, *Il17f*^*–/–*^or *Il17af*^*–/–*^mice. Single-cell suspensions were prepared from peripheral lymph nodes (pLN) and spleen and enriched via magnetic microbeads (MACS, CD4^+^ T cell isolation kit; Miltenyi Biotec, Bergisch-Gladbach, Germany). BM cells were harvested from the femurs and tibias of donor mice. BM cells were stained with biotinylated anti-CD3 (clone 17A2, homemade) and separated via streptavidin-conjugated magnetic beads (Miltenyi Biotec, Bergisch-Gladbach, Germany) in order to deplete T cells. After transplantation, mice were kept on antibiotic water (Cotrimoxazol; Ratiopharm, Ulm, Germany) until the end of the experiment. Survival, weight loss and clinical GVHD-signs of recipient mice were monitored and scored according to Cooke et al [21]. Clinical signs of acute GvHD, such as ruffled fur, weight loss (mild >10% of initial body weight; severe >25% of initial body weight), hunched back, inactivity and diarrhea, were monitored two times per day. Severity of each clinical sign was scored (no = 0; mild = 1; severe = 2) and animals with a total score of ≥6 were sacrificed immediately by cervical dislocation and counted as GVHD lethality. All transplanted mice were provided with moistened food to allow easier feeding and aid hydration. Hannover Medical School provided the research staff with special training in animal handling. Despite frequent monitoring, occasionally, mice were found dead without clinical signs of GVHD within the first 10 days after transplantation. This was limited to fewer than 5% of mice involved in the study and was considered as non-GVHD mortality, and those mice were excluded from the final analysis.

### Cell proliferation assay

Proliferation of *Il17af*^*–/–*^or WT T cells after unspecific stimulation with CD3/CD28 beads (ThermoFisher, Schwerte, Germany) or allogeneic BM-derived DCs was determined by ^3^H-Thymidine uptake as follows: 5,000 cells were incubated in a 96-well plate with 150μl RPMI medium supplemented with 10% FCS, 1% L-glutamine, 1% Pen-Strep and 0.04% gentamycine. The cells were incubated for two days in 95% humidified atmosphere by 5% CO_2_ at 37°C, before 0.8 mCi ^3^H-Thymidine (Hartman) was added per well. The incorporation of radioactive thymidine was measured 16h later in a Microbeta workstation (Perkin Elmar).

### BrdU proliferation assay

Day 20 after BMT, mice were i.p. injected with 3mg 5-Bromo-2′-deoxyuridine (BrdU) (Sigma-Aldrich) and set on 0.8mg/ml BrdU containing water over night. After 24h, mice were sacrificed 24h and SI and colon were fixed in 4% formaline (Sigma-Aldrich) overnight and processed as described above. Detection of incorporated BrdU was performed with the BrdU In-Situ Detection Kit (Cat No. 550803, BD Biosciences) according to the manufacturer’s manual. Pictures were acquired with an Olympus BX61 (Olympus, Hamburg, Germany) confocal microscope and processed with the cellSens Dimensions 1.9 software (Olympus, Hamburg, Germany).

### 16S DNA Illumina sequencing of stool bacteria in GVHD situation

Fresh stool samples were collected from *il17af*^*–/–*^and WT recipients at day 14 and day 21 after BMT. Bacterial DNA was isolated using QIAamp DNA Stool Mini Kit (Qiagen) according to manufacturer’s manual. In the first PCR round, 16S V3-V4 regions were amplified by 15 PCR cycles using published primers [22]:

S-D-Bact-0341F (5’-acactctttccctacacgacgctcttccgatctCCTACGGGNGGCWGCAG-3’) and S-D-Bact-0785R (5’-gtgactggagttcagacgtgtgctcttccgatctGACTACHVGGGTATCTAATCC-3’). In the second PCR with 13 cycles Illumina-adapters were added using following primers: adapter_for: (5’ aatgatacggcgaccaccgagatctacactctttccctac 3’) and adapter_rev: (5’-caagcagaagacggcatacgagatXXXXXXgtgactg-3’). The XXXXXX bases represent the MID region, each sample was coded with a distinct DNA fragment for later assignment. All PCR steps were performed with Advantage2 PCR kit (Takara). PCR products were separated with a 2% agarose gel in TAE buffer. For gel extraction, QIAquick Gel extraction kit (Quagen) was used according to manufacturer’s manual. Library concentration was adjusted and 250bp paired-end sequencing was performed on the Illumina MiSeq system following standard protocol. Quality control and adapter clipping of the sequences was done using fastq-mcf tool of ea-utils [23]. Sequencing data were processed according to the workflow listed: Paired-reads were joined using of ea-utils. Chimeras were excluded using Usearch [24] sequence analysis tool with uchime [25] command based on chimeraslayer gold 16s rRNA database (release 4.29.2010) as reference. Taxonomy assignment was performed by RDP-classifier 2.8 [26] with confidence value of 0.5.

### Transendothelial electrical resistance (TEndoR)

Human umbilical vein endothelial cells (HUVECs) were grown to confluence in polycarbonate wells containing evaporated gold microelectrodes in series with a large gold counter connected to a phase-sensitive lock-in amplifier as described previously. TEndoR was measured using an electrical cell-substrate impedance sensing system (ECIS) (Applied BioPhysics Inc.) as described elsewhere [27]. Each condition’s endpoint resistance was divided by its starting resistance to give the normalized TEndoR. When the cells reached the resistance more than 1500 ohm, cells were treated with 10ng/ml IL-17 (Sigma-Aldrich, St-Luis, MO) and TEndoR was measured thereafter in real-time.

### Transepithelial resistance (TEpiR) and fluorescein permeability measurements

Epithelial permeability: Caco-2Bbe cells were seeded on 0.4 μm snapwells (Corning Life Sciences, USA) at 1.5 × 104/cm^2^ and grown for 14 days. TEpiR was measured daily using a EVOM2 voltohmmeter (World Precision Instruments, Sarasota, FL). On day 14, IFN-γ 10ng/ml (Sigma Aldrich, Germany) ±IL-17 (IL-17A or IL-17F 2.5ng/ml ~ 10ng/ml) (Sigma Aldrich, Germany) was added to the apical and basolateral bath every 12 hours. After 24h, both sides of the cell monolayer were incubated with TNF-α 10ng/ml ± IL-17 (IL-17A or IL-17F 2.5ng/ml ~ 10ng/ml) in the continuous presence of INF-γ, and TEpiR was measured before, 2h, 4h, 8h, 12h and 24h after TNF-α addition. Subsequently, the cell were transferred to an Ussing Chamber system (Easymount, Physiologic Instruments, CA), incubated in a buffer containing NaCl 116 mM, KH2PO4 0.4 mM, K2HPO4 2.4 mM, MgCl2 1.2 mM, CaCl2 1.2 mM, NaHCO3 24 mM, gassed with 95% O2 and 5% CO2 at 37°C. TEER was monitored with KCl agar electrodes. 100 μMfluorescein or (FITC)-dextran-4000 was added to the apical side, and both apical and basolateral samples were collected 1 hour later and measured with a fluorometer at 520 nm (Tecan Infinite M200, Tecan, Switzerland). Fluorescein and (FITC)-dextran-4000 paracellular fluxes were presented as the ratio of the tracer in the basolateral compartment and in the apical compartment. Tracer fluxes = basolateral FITC intensity / apical FITC intensity × 100%. Data are presented as mean ± SEM, n = 3–4 samples in each condition.

### Data analysis and statistics

Statistical analysis was performed with Prism 7 (Graph-Pad Software, Inc.). Statistical differences for the mean values are as follows: *, P≤0.05; **, P≤0.01; and ***, P≤0.001. Student’s t test, Mann Whitney U test or ANOVA were used for calculating statistical significance. The analysis of survival data was performed using Kaplan-Meier estimation and log-rank test.

Further details on methods are available as supplementary methods and material.

## Results

### Donor IL-17A and IL-17F deficiency leads to aggravated acute GVHD

**Fig 1:**
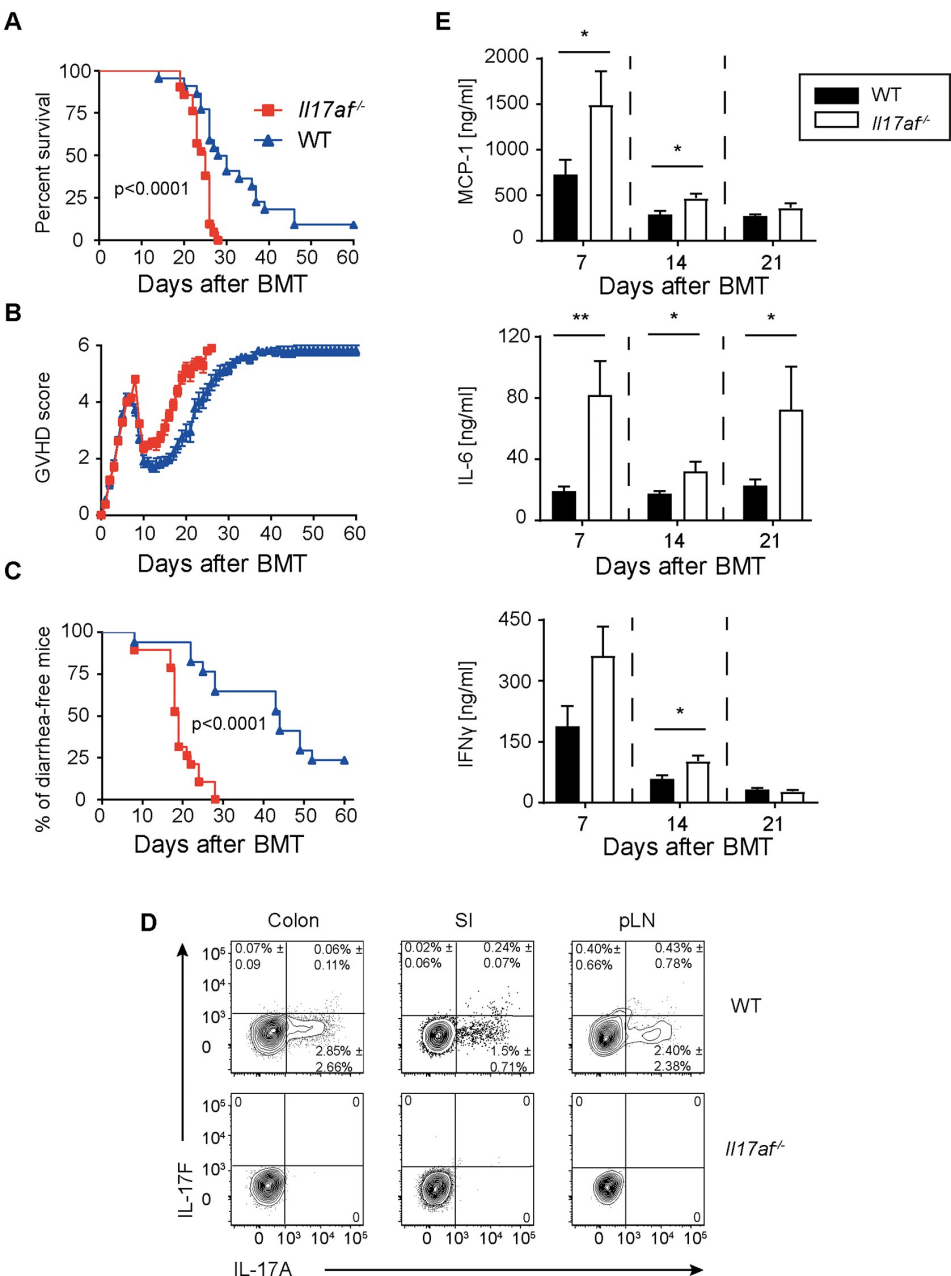
Deficiency of IL-17A and IL-17F in donor CD4^+^ T cells leads to aggravated GVHD. BALB/c mice were lethally irradiated and transplanted with 5x10^6^ TCD BM and 0.5x10^6^ CD4^+^ T cells from BL6 WT or *Il17af*^*-/-*^ donors. A) Survival curve of *Il17af*^*-/-*^ and WT T cell recipients. Data are pooled from four independent experiments (*Il17af*^*-/-*^ n = 21, WT CD4^+^ cells n = 22). For statistical analysis the log rank test was used. B) Clinical score. C) Percentage of diarrhea-free mice. D) FACS sorted donor Thy1.1^+^ CD4^+^ T cells were analyzed for the expression of IL-17A and IL-17F. Donor WT or *Il17af*^*-/-*^ CD4^+^ T cells were isolated from BALB/c recipients from colon, SI and pLNs on day 21 after BMT. Data were collected from three independent experiments for colon and SI (WT n = 10, *Il17af*^*-/-*^ n = 11); and two experiments pLNs (WT = 8, *Il17af*^*-/-*^ n = 7). E) Concentrations of IL-6, MCP-1 and IFNγ cytokines in the sera of WT or *Il17af*^-/-^ CD4^+^ T cell recipients sacrificed at day 7, 14, and 21 after BMT. (day 7 WT n = 10, *Il17af*^-/-^n = 10; day 14 WT n = 11, *Il17af*^-/-^n = 12; day 21 WT n = 16, *Il17af*^*-/-*^ n = 15). Statistical significance was determined by Student’s *t* test. The bars show the mean and error bars show SEM.

To assess the effects of IL-17 produced by donor-derived Th17 cells in acute GVHD, we compared the outcome of experimental acute GVHD induced by adoptive transfer of allogeneic CD4^+^ T cells from wild type (WT) or *Il7af*^*-/-*^ donors [5] using C57BL/6 donors and lethally irradiated BALB/c recipients. Phenotype of steady state CD4^+^ cells from *Il17af*^-/-^ mice were analyzed by flow cytometry and showed no major differences compared to WT CD4^+^ T cells (S1 Fig). Recipients of *Il17af*^-/-^ CD4 T cells showed a significant increase of GVHD severity and mortality as compared to WT controls (Fig 1A and 1B). Interestingly, recipients of *Il17af*^*-/-*^ T cells suffered from severe diarrhea early after transplantation (Fig 1C).

**Fig 2:**
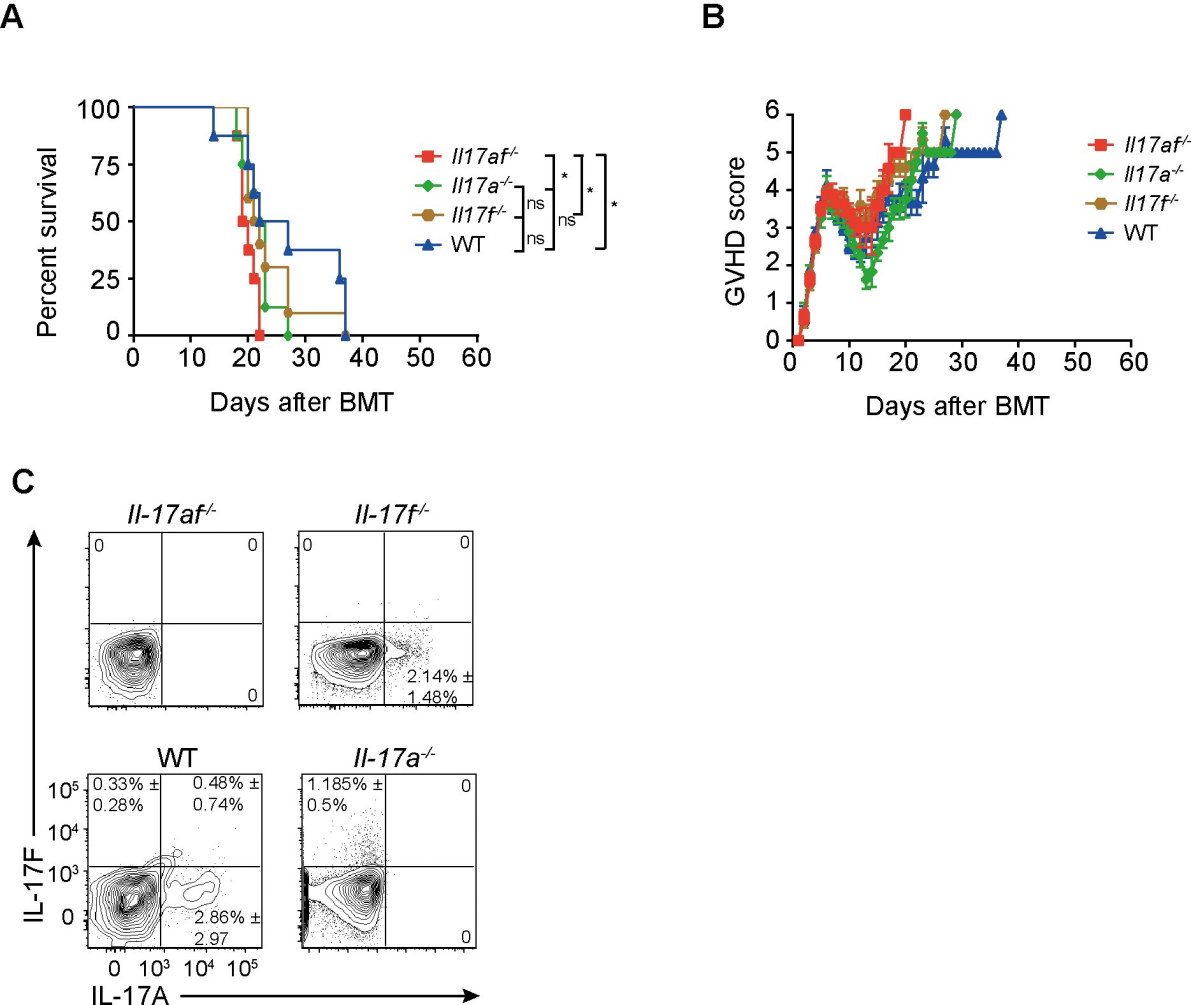
IL-17A and IL-17F are reciprocally compensated during GVHD. BALB/c mice were lethally irradiated and transplanted with 5x10^6^ TCD BM and 0.5x10^6^ CD4^+^ T cells from BL6 WT, *Il17af*^-/-^, *Il17a*^-/-^, or *Il17f*^-/-^ donors. A) Survival curve of BL6 BL6 WT, *Il17af*^-/-^, *Il17a*^-/-^, or *Il17f*^-/-^ CD4^+^ T cell recipients, data were generated in two experiments (BL6 WT, *Il17af*^-/-^, *Il17a*^-/-^n = 8, *Il17f*^-/-^ n = 10) B) Clinical score. C) Expression of IL-17A and IL-17F by donor CD4^+^ T cells isolated from pLNs of BL6 WT, *Il17af*^-/-^ *Il17a*^-/-^, or *Il17f*^-/-^ CD4^+^ T cell recipients in experimental GVHD at day 21 after transplantation. For statistical analysis, non-parametric two-tailed T test was used. Data represent the frequency and the SD of a single experiment (n = 3 per group). *p≤0.05.

To verify the occurrence of Th17 cells after BMT, we analyzed IL-17 secretion of CD4^+^ T cells in host tissues by intracellular cytokine staining. We re-isolated donor lymphocytes from recipients’ colon, small intestine (SI) and lymph nodes 21 days after transplantation and stained for IL-17A and IL-17F. Thy1.1 was used to separate donor from remaining host CD4^+^ T cells that escaped elimination by conditioning. IL-17A and F secretion by CD4 T cells was evident in peripheral lymph nodes (pLN) and GVHD target organs **(**Fig 1D). Next, we analyzed systemic cytokine levels after GVHD initiation. We observed a marked increase of IL-6, IFNγ and MCP-1 levels in *Il17af*^-/-^ recipients as compared to WT-recipients at different time points after transplantation (Fig 1E). From these data we conclude that donor-derived IL-17A and IL-17F exert protective effects in the early course of GVHD. Since the IL-17 isoforms A and F both bind to the IL-17RA receptor, we checked whether either lack of IL-17A or F altered protection from acute GVHD. To that end, we made use of single *Il17a*^-/-^, *Il17f*^-/-^ and double IL-17-deficient (*Il17af*^-/-^) CD4^+^ T cells. We observed that only recipients receiving *Il17af*^-/-^, but not *Il17a*^-/-^, *Il17f*^-/-^ CD4^+^ T cells, developed an aggravated GVHD as compared to WT controls (Fig 2A and 2B). In order to validate our experimental setup, we re-isolated donor CD4^+^ T cells from pLNs of recipients and stained for IL-17A and IL-17F to check for secretion of IL-17 cytokines. Indeed, we observed expression of both IL-17A and IL-17F from WT CD4^+^ cells, whereas *Il17a*^-/-^ and *Il17f*^-/-^ CD4^+^ T cells expressed only IL-17F and IL-17A respectively. Expectedly, *Il17af*^-/-^ CD4^+^ T cells did neither express IL-17A nor IL-17F (Fig 2C).

### IL-17 deficiency of donor T cells does not alter the microbial flora after BMT

**Fig 3:**
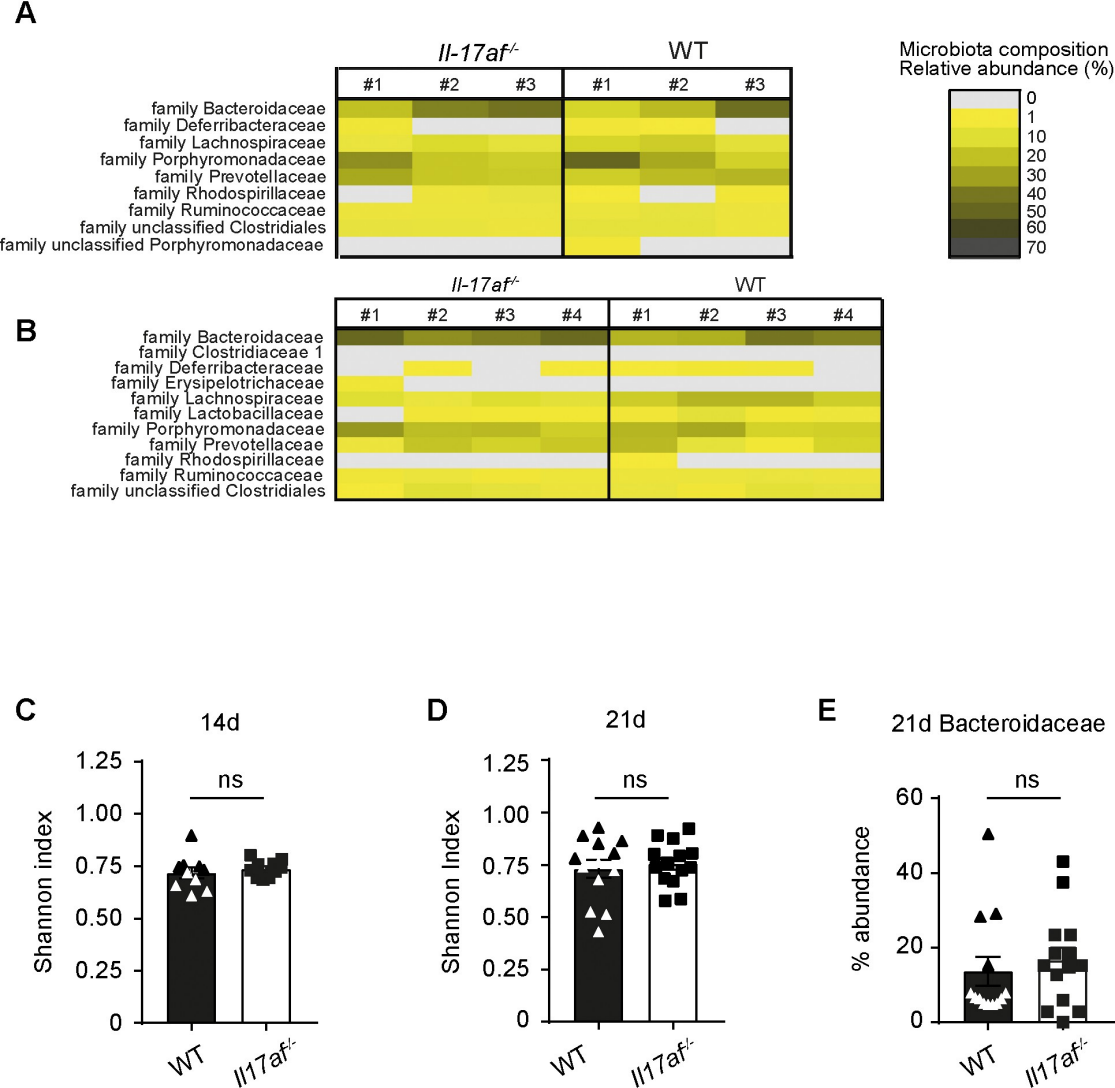
IL-17 A/F deficiency does not alter the microbial flora after allogeneic BMT. BALB/c mice were lethally irradiated and transplanted with 5x10^6^ TCD BM and 0.5x10^6^ CD4^+^ cells from BL6 WT or *Il17af*^-/-^donors. A-B) Representative graph of microbial flora in the feces of WT and *Il17af*^-/-^T cell recipients at A) day 14 or B) day 21 after BMT. Heat-maps of the relative abundance of bacterial species at phylogenetic level of families are presented for one representative experiment. Each column represents one sample from individual mouse. (day 14 WT n = 3, *Il17af*^-/-^n = 3; day 21 WT n = 4, *Il17af*^-/-^n = 4). C) Shannon index of diversity for all samples on the family level at day 14 and D) day 21 after BMT. E) Relative abundance of Bacteroidaceae family in the feces of WT and *Il17af*^-/-^ T cell recipients at day 21 after BMT. (day 14 WT n = 10, *Il17af*^-/-^n = 10; day 21 WT n = 13, T *Il17af*^-/-^n = 14). Sequences with an abundance of less than 1% were excluded from the analysis.

The composition of the gut microbiome affects GVHD outcome [28] and it has been shown that Th17 cells have an impact on modulation of the microbiome in several experimental GVHD models [29]. Of note, *bacteroidaceae* species have been shown to compose the majority of the stool microbiota in patients without acute GVHD [30]. Therefore, we sequenced stool samples of *Il17af*^-/-^ or WT CD4^+^ T cell recipients at day 14 and day 21 day after transplantation (Fig 3A and 3B). Interestingly, we found no significant difference in the overall microbiota diversity between recipients either on day 14 or day 21 post allo-BMT as assessed by Shannon indices (Fig 3C and 3D) or in prevalence of the *bacteroidaceae* family between groups (Fig 3E).

### Lack of donor CD4^+^ T cell-derived IL-17A and IL-17F results in increased intestinal leakage

**Fig 4:**
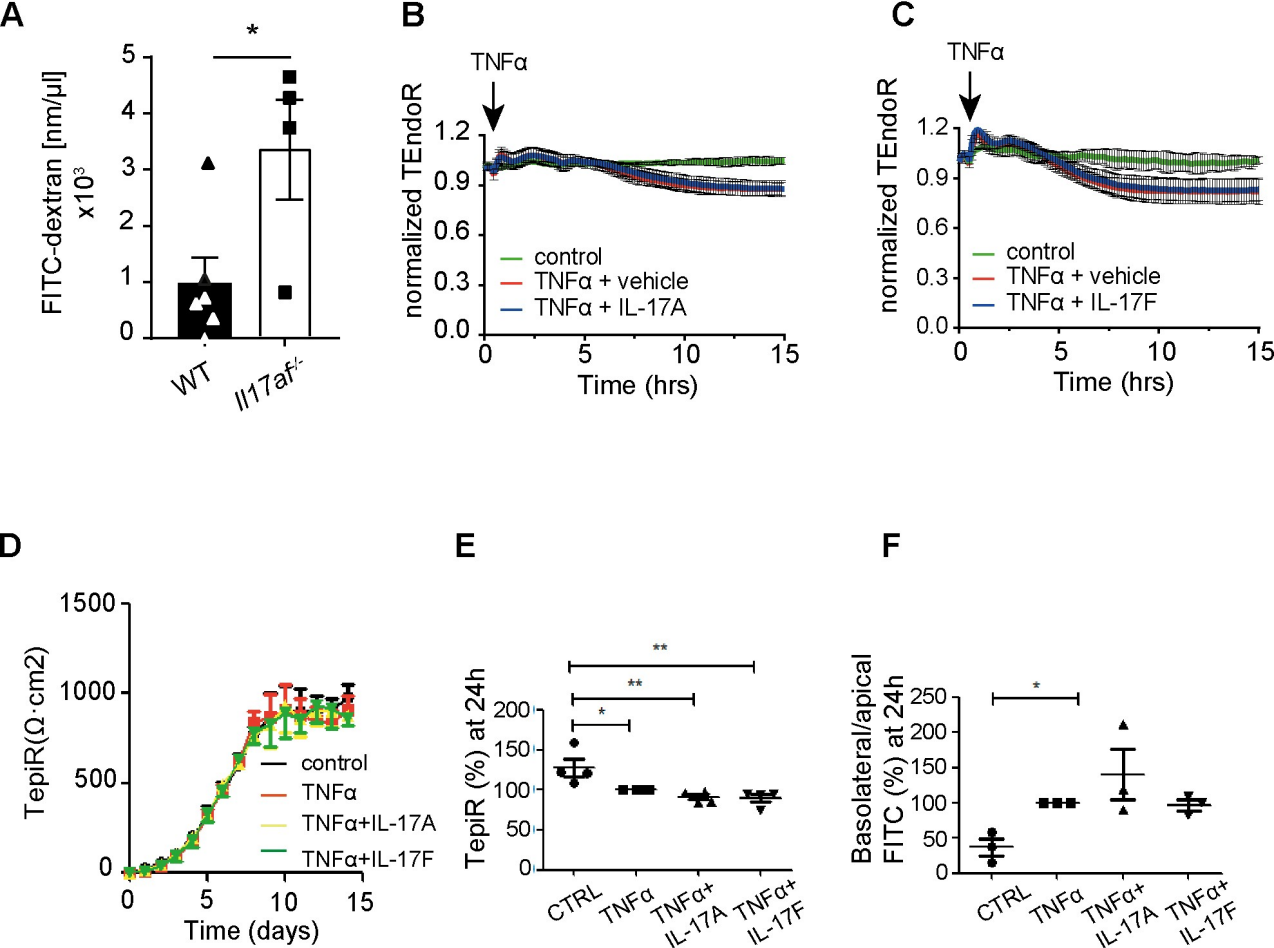
Recipients of *Il17af*^-/-^ CD4^+^ cells show gut-leakiness. BALB/c mice were lethally irradiated and transplanted with 5x10^6^ TCD BM and 0.5x10^6^ CD4^+^ cells from BL6 WT or *Il17af*^-/-^ donors. A) Concentration of translocated FITC-dextran in the sera of WT and *Il17af*^-/-^ CD4^+^ T cell recipients at day 21 after BMT. (n = 6, *Il17af*^-/-^ n = 4) B) Increase in transendothelial electrical resistance (TEndoR) normalized to vehicle. C) Increase in transepithelial electrical resistance (TepiR, measured daily with an EVOM2 voltohmmeter, over the days in culture after confluency. All four groups had a similar increase in TEpiR prior to the cytokine treatment. D) at day 14 post confluency, the cells were incubated with 10ng/ml interferon-γ ± IL17A or IL17F, followed by 10g/ml TNF-α 10ng/ml ± IL-17 (IL-17A or IL-17F 10ng/ml) 24 h later, and TEpiR was assessed at the indicated times. E) After 24h, the filters were transferred to an Ussing-chamber system, and both TEpiR, and F) FITC permeability was assessed. The values in C and D are given in % of the value treated with IFN-γ and TNF-α only. All data are pooled from two or three independent experiments. Error bars represent SEM. For statistical analysis Log-rank test was used. *p≤0.05.

Recipients of *Il17af*^-/-^ CD4^+^ T cells showed early and severe diarrhea (Fig 1C), suggesting alteration of the intestinal barrier. To test the functionality of the intestinal barrier after GVHD-initiation, we applied FITC-dextran by oral gavage to BMT-recipients [31]. Thereafter, serum FITC-Dextran concentrations were assessed at day 21 post-transplantation. We observed a significantly increased intestinal leakage in recipients of *Il17af*^-/-^ CD4^+^ T cells (Fig 4A), indicating intestinal barrier breakdown. We tested whether IL-17 cytokines exert protective effects *in vitro*. For this purpose, we used ECIS to analyze the effect of IL-17 on endothelial leakage. Interestingly, we did not see any effect of either of the IL-17 cytokines in reduction of TNFα- induced leakage in such experiments (Fig 4B).

There has been emerging evidence of a protective role of IL-17 in fostering gut integrity via modulation of epithelial tight junctions, albeit in a different experimental setup [32]. Therefore, we analyzed the consequences of IL-17A and IL-17F administration in an *in vitro*-system for gut-epithelial-leakage. To that end, we used the well-characterized Caco2BBe cell line expressing IL-17 receptor, grown as differentiated monolayer cultures on permeable filters, and measured the TEpiR as a measure for paracellular permeability. TEpiR was measured daily with an EVOM2 (Voltohmmeter, WPI), and at the end of the incubation time in an Ussing chamber setup. Additionally, we assessed apical to basolateral fluorescein (FITC) fluxes. However, we saw no evidence of a protective role of IL-17A or IL-17F against TNFα-induced decrease in TEpiR (Fig 4C and 4D). While TNFα incubation resulted in a significant decrease in TEpiR, verified by TEpiR measurements in the Ussing Chamber (Fig 4E) and by an increase in FITC permeability, the pre-incubation for 24 h with IL-17A or IL-17F did not prevent the increase in epithelial permeability (Fig 4F).

### Lack of donor-derived IL-17A and F cells leads to increased IFNγ production of donor CD4 T cells and macrophage influx to the intestine

**Fig 5:**
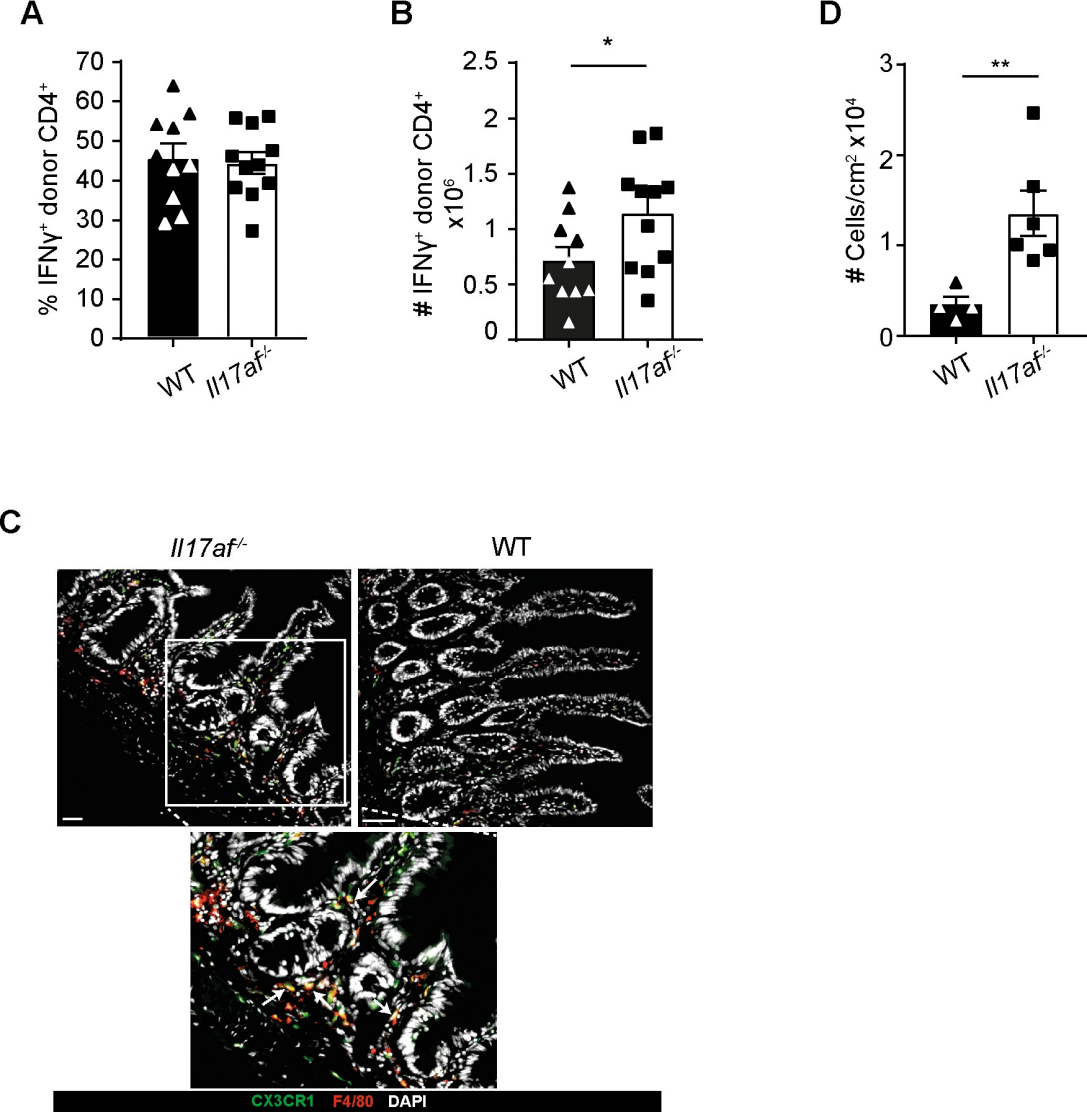
Lack of IL-17 causes CD4^+^IFNγ^+^ and macrophage infiltration. BALB/c mice were lethally irradiated and transplanted with 5x10^6^ TCD BM and 0.5x10^6^ CD4^+^ cells from BL6 WT or *Il17af*^-/-^donors. A) FACS sorted donor Thy1.1^+^ CD4^+^ T cells from the SI of the BALB/c mice stained for the expression of the IFNγ on day 21 post BMT. Gating was performed on donor lymphocytes using Thy1.1 as congenic marker. Each dot represents the total amount of cells isolated from the organ of a single mouse; the bars show the mean (WT n = 10; *Il17af*^-/-^ n = 11). B) Representative staining for macrophage infiltrates in the small intestine of BALB/c recipients on day 21 after BMT. Arrows indicate DAPI^+^CX3CR1^+^F4/80^+^ cells. Scale bars represent 50μm C) Absolute number of DAPI^+^CX3CR1^+^F4/80^+^ cells per cm^2^. (WT n = 4; *Il17af*^-/-^ n = 6) All data are pooled from two or three independent experiments. Error bars represent SEM. For statistical analysis, non-parametric two-tailed T test was used. *p≤0.05; **p≤0.01.

Th1 T cells producing the proinflammatory cytokine IFNγ have been shown to be detrimental in inducing GVHD [33]. Hence, we compared the numbers of CD4^+^ T cells secreting IFNγ in recipients of *Il17af*^-/-^ and WT CD4 T cells. Interestingly, the percentage of IFN γ^+^ CD4 T cells did not change significantly (Fig 5A). However, we detected increased numbers of CD4^+^IFNγ^+^ T cells in the intestine of *Il17af*^-/-^recipients (Fig 5B). These results show that lack of donor Th17 leads to a Th1 shift within the CD4^+^ T cell compartment. Along the same lines, IFNγ promotes macrophage activation [34], therefore we analyzed macrophage-infiltration to the gastrointestinal tract. To that end, we sacrificed the mice 14 and 21 days after BMT and checked for macrophage infiltration in the intestine using a CX3CR1-GFP reporter mice as BM donors (Fig 5C). Indeed, we observed a significantly greater number of infiltrates in SI of animals receiving *Il17af*^-/-^ CD4 T cells (Fig 5D).

### Small intestine epithelial proliferation is impaired in Il17af^-/-^ CD4^+^ recipients

**Fig 6:**
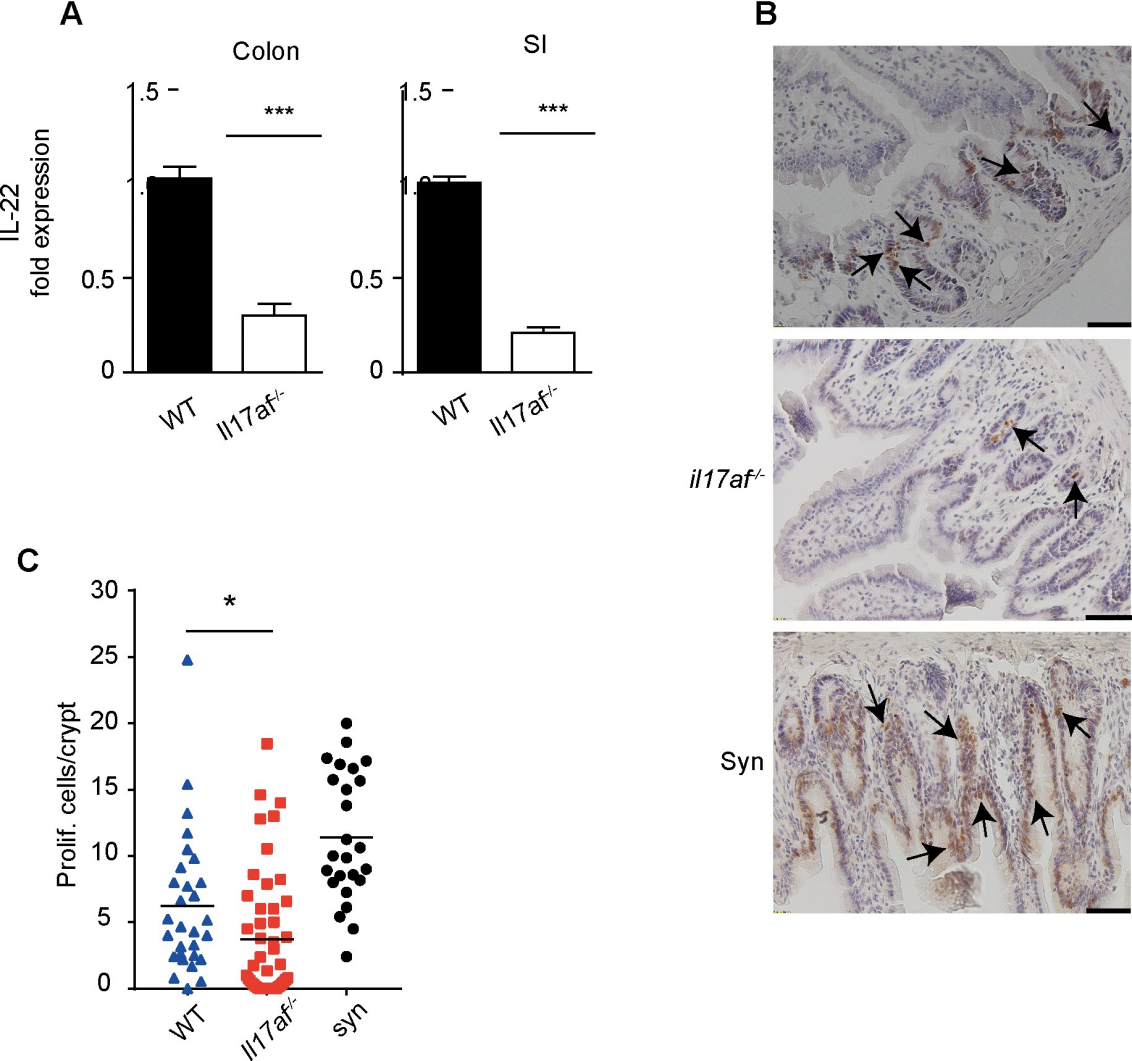
IL-17 deficiency in donor CD4^+^ cells leads to impaired epithelial proliferation. A) Relative expression level of IL-22 mRNA in colon and SI at day 14 after BMT. The expression level of IL-22 mRNA in *Il17af*^-/-^ CD4^+^ T cell recipients was calculated in relation to WT CD4^+^ T cell recipients. Expression of target genes was normalized to the expression of housekeeping genes HPRT or GAPDH (WT n = 6; *Il17af*^-/-^ n = 6). B) BALB/c recipients of *Il17af*^*-/-*^ or WT CD4^+^ T cells were treated with BrdU i.p. at day 20 and sacrificed at day 21 after BMT Representative BrdU incorporation in the SI at day 21 after BTM. Scale bars represent 50μm and brown cells (indicated by black arrows) are BrdU incorporating cells. C) Number of proliferating intestinal epithelial cells per crypt, marked by BrdU incorporation. The black lines represent the mean value and the dots indicate the number of BrdU^+^ cells per crypt (WT n = 7, *Il17af*^*-/-*^ n = 8, syn n = 5). Cells were counted in blinded manner. All error bars show SEM. All data are pooled from two or three independent experiments A-C. For statistical analysis, non-parametric two-tailed T test and one-way ANOVA were used. *p≤0.05; ***p≤0.001.

IL-22 is a homeostatic cytokine secreted by Th17 cells, which can preserve the integrity of the mucosal tissues [35]. A protective role of IL-22 in GVHD via conservation of intestinal stem cells has been demonstrated [31]. Consequently, we analyzed IL-22 expression in the gastrointestinal tract (SI and colon) in recipients of *Il17af*^-/-^and WT CD4^+^ T cells two weeks after transplantation. IL-22 expression levels were significantly lower in recipients of IL-17 deficient cells (Fig 6A). These observations suggest that the integrity and possible proliferation of intestinal stem cells was impaired in *Il17af*^-/-^ cells recipients. Therefore, we analyzed the numbers of proliferating intestinal epithelial cells per crypt. We found a significantly lower number of proliferating cells in in recipients of *Il17af*^-/-^ CD4^+^ T cells, indicating reduced recovery after local tissue damage (Fig 6B and 6C).

## Discussion

Contribution and exact function of each of the IL-17 cytokines in health and disease have been a topic of extensive research for the last decade [36]. Much of the difficulty in characterizing exact roles of IL-17 cytokine responses lies in the plasticity of the Th17 cells involved in the response [18]. Furthermore, despite numerous efforts, there has been conflicting evidence of the individual contribution of IL-17A and IL-17F cytokines to the pathology of acute GVHD. In this study we provide evidence of exacerbated acute GVHD and consequently increased mortality in mice transplanted not only with single IL-17A, but with double deficient *Il17af*^-/-^ CD4^+^ T cells. A protective role of IL-17 in GVHD development in a similar model has been previously reported by Yi et al [16]. In line with those results we propose a mechanism of aggravated Th1 responses by IL17-deficient CD4^+^ T cells, resulting in aggravated GVHD.

Both IL-17A and IL-17F can be bound by IL-17RA/RC dimer [8]. Even though the two cytokines share high homology at the amino acid level [37], their functional differences remained ambiguous, with recent research pointing to different contributions of each cytokine to colitis pathology [38]. However, in our model only recipient mice transplanted with cells deficient for both cytokines exhibited a statistically significant increase in mortality, whilst we only observed a trend of increased mortality in recipients transplanted with *Il17a*^-/-^ or *Il17f*^-/-^ CD4^+^ T cells. Despite the previously published data of similar, but distinct roles of IL-17A and IL-17F [10,11], we found no evidence of differential effects of the two cytokines in our model. Therefore, our data points to potential redundancy of IL-17A and IL-17F in the pathology of acute GVHD. However, other groups have shown evidence of potentially distinct roles as being model-dependent [12]. Due to the severity of the model, it is likely that we cannot see minor differences due to profound tissue damage and severe systemic inflammation. Therefore, further research is needed to study distinct roles of each IL-17 cytokine in the context of GVHD.

The exacerbated GVHD in recipients of *Il17af*^-/-^ CD4^+^ cells raise the question of origin of IL-17 cytokines regulating the disease progression. Several subtypes of immune cells are capable of IL-17 production. For instance, IL-17^+^γδ T cells have been demonstrated by many groups as potent inflammation initiators [39]. Similarly, another possible source of IL-17 might be innate lymphoid cells [40,41]. Therefore, even though unlikely, one cannot fully exclude the possibility of other IL-17 secreting cells in our experimental system. We found the exacerbated gastrointestinal GVHD, in our model was driven by IL-17 cytokines from the donor CD4^+^ T cells. This is in line with published literature describing a role of donor-derived IL-17 in gut GVHD [16], but in contrast with research done by Kappel et al. which showed an ameliorated GVHD in the absence of donor IL-17A, albeit using a different experimental setup [15]. On the other hand, recipient-derived IL-17 might also play a role in the modulation of GVHD, even independent of microbiome modulation as indicated by Varelias et al.[29].

In the past few years, the connection between microbiota diversity and occurrence of GVHD has been studied intensively [28]. Of note, a recent study demonstrated the importance of maintenance of *bacteroidaceae* family after BMT, and IL-17 was intricately connected with changes to the microbiota in patients [30]. However, we did not observe any differences in the microbiota composition nor prevalence of the *bacteroidaceae* family in our study. The reason for this apparent discrepancy is likely due to the fact that most of the previously published data reporting the link between Th17 cells and microbiome in patients has identified recipient-derived IL-17 as the driving factor of microbiota changes [29,38]. On the other hand, here we have identified the lack of IL-17A and Il-17F production by donor CD4^+^ T cells as one of the factors exacerbating the disease. However, one cannot fully exclude a possible effect of IL-17- secreting cells on the changes in the microbiota in GVHD context. Using different models and further research is warranted to elucidate this complex link.

IFNγ is a well-known potent driver of Th1 response [34] and the balance between Th1 and other CD4 T cell subsets, such as Th17 seems to be an important factor in determining GVHD outcome. However, the exact role of IFNγ in GVHD is to this day a topic of debate with overwhelming evidence of gut-GVHD promotion by donor-derived T cells [42,43]. In this study we observed a significant increase of both IFNγ in the blood sera and increased numbers of CD4^+^IFNγ^+^ cells in the SI, as well as severe diarrhea of the recipient transplanted with *Il17af*^-/-^ cells. Those observations led us to believe that there is a local gut tissue pathology underlying aggravated GVHD. Indeed, we could show that mice transplanted with *Il17af*^-/-^ CD4^+^ cells appeared to have a leakier gut, compared to those transplanted with WT CD4^+^ cells, as shown by the FITC-dextran translocation from the gut to the blood sera. However, this apparent local tissue damage raises the question whether it is a direct effect of the IL-17 cytokines deficiency on either the epithelium or endothelium, or is it an indirect effect driven by the immunity switch to Th1 response. It has been hypothesized that IL-17 regulates intestinal epithelial permeability via the tight-junction protein occludin [32]. However, we saw no direct protective effect of either of the IL-17 cytokines in our *in vitro* models for endothelial and epithelial permeability. This data pointed in the direction of an indirect effect of donor-derived IL-17 in the pathophysiology of acute GVHD.

Macrophages are a part of the innate immunity and one of the main effector cells of the Th1 response [44], especially in the context of acute GVHD [45,46]. We speculate that the macrophages found in our model respond to the IFNγ secreted by the donor CD4^+^ cells and aggravate local tissue damage in the intestinal tract. This is likely not the only cause of extensive damage to the gastrointestinal tract, and we believe this effect to be further exacerbated by inability of the gut to restore its function, indicated by reduced proliferation of intestinal epithelial cells in the *Il17af*^-/-^ CD4^+^ recipients.

Taken together, our data confirms a beneficial role of IL-17 cytokines in maintenance of balance of Th1 and Th17 responses in acute GVHD. Transplantation of allogeneic *Il17af*^-/-^ CD4^+^ T cells generates a high frequency of CD4^+^IFNγ^+^ T cells and macrophage infiltration into the intestinal tract. Furthermore, gut stem cell recovery is affected in the absence of donor-derived IL-17A and F. Such response results in aggravated acute GVHD. In conclusion, IL-17 cytokines plays a major, however non-binary role following allo-BMT and its selective targeting may be of considerable translational value in preventing severe acute gastrointestinal GVHD.

## Supporting information

S1 Fig *Il17af*^-/-^CD4^+^ cells display a similar activation phenotype to WT CD4^+^ T cells. Treg proportions and activation profile are shown from spleenocytes collected from either WT or Il17af-/- mice at steady-state conditions. Data are pooled from two independent experiments. For statistical analysis, non-parametric two-tailed T test and oneway ANOVA were used. *p≤0.05.
(PDF)

S1 File (DOCX)
