# Supplementary material for: Donor-derived IL-17A and IL-17F deficiency triggers Th1 allo-responses and increases gut leakage during acute GVHD
Source: PLoS One. 2020 Apr 6;15(4):e0231222. doi: 10.1371/journal.pone.0231222 (PMC7135231; doi:10.1371/journal.pone.0231222)
Supplement: S1 Fig — Treg proportions and activation profile are shown from spleenocytes collected from either WT or Il17af-/- mice at steady-state conditions. Data are pooled from two independent experiments. For statistical analysis, non-parametric two-tailed T test and oneway ANOVA were used. *p≤0.05. (PDF) [file pone.0231222.s001.pdf]

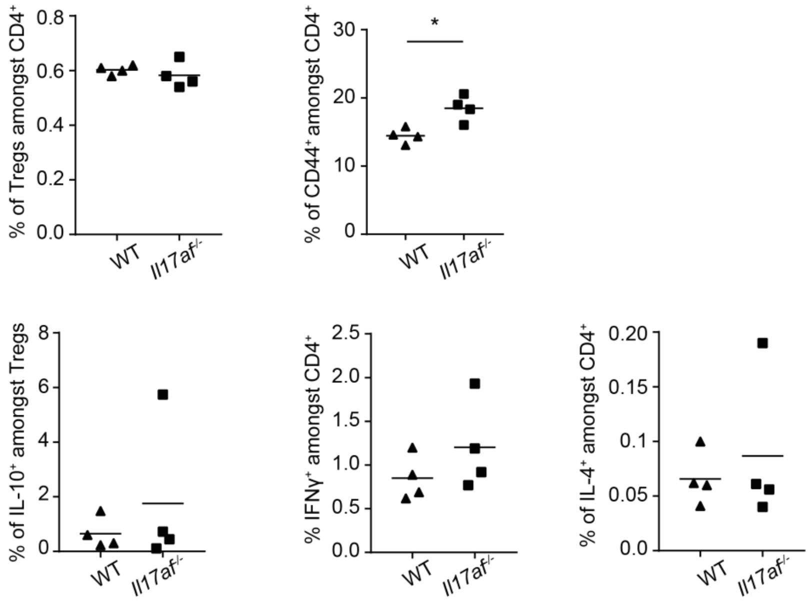

Supplementary Figure 1: Il17af$^{-/-}$CD4^{+} cells display a similar activation phenotype to WT CD4^{+} T cells. Treg proportions and activation profile are shown from spleenocytes collected from either WT or Il17af$^{-/-}$ mice at steady-state conditions. Data are pooled from two independent experiments. For statistical analysis, non-parametric two-tailed T test and one-way ANOVA were used. *$p \leq 0.05$
