# Supplementary material for: Donor-derived IL-17A and IL-17F deficiency triggers Th1 allo-responses and increases gut leakage during acute GVHD
Source: PLoS One. 2020 Apr 6;15(4):e0231222. doi: 10.1371/journal.pone.0231222 (PMC7135231; doi:10.1371/journal.pone.0231222)
Supplement: S1 File — (DOCX) [file pone.0231222.s002.docx]

**Supplementary Material and Methods**

*Isolation of donor T cells after BMT*

To determine the infiltration of donor lymphocytes into GVHD target organs, recipient mice were sacrificed at different time points after BMT and secondary lymphoid organs (SLOs), small intestine (SI), colon and lungs were harvested. Isolation of infiltrating cells was performed as described (1). For isolation of lung infiltrating T cells, lungs were cut into small pieces and incubated in RPMI 1640 medium supplemented with 10% FCS, 20mM HEPES, 0.5mg/ml collagenase D and 0.025mg/ml DNAse I (Sigma-Aldrich, St-Luis, MO) for 45 min at 37°C. The digested tissue was mashed through a nylon mesh and washed with PBS containing 3% FCS. Subsequently the flow-through was pelleted, suspended in RPMI 1640 medium with 10% FCS and lymphocytes were recovered by Lympholyte M (Cedarlane Labs, Paletta, Kanada) gradient centrifugation.

*Antibodies and Flow Cytometry*

The following antibodies were purified from hybridoma supernatants and conjugated in house: anti-Gr1 Cy5 (Ly-6G), anti-CD8b Cy5 (RmCD8), anti-CD4 Alexa 488 (RmCD4-2). Anti-CD4 PerCP (RM4.5) and anti-CD3 PE (17A2) antibodies were purchased from BioLegend, San Diego, CA). Anti-CD8a (53-6.7) and anti-Thy1.1 PE (CD90.1) were purchased from BD Pharmingen, São Paulo, Brazil). Anti-CD44 APC (IM7) was purchased from eBioscience, San Diego, USA). The labeling of living cells was performed in PBS supplemented with 3% FCS after blocking with 10% CD16/32 Fc block (2.4G2, homemade). For intracellular stainings, single cell suspensions of lymphoid cells were stimulated with 50 ng/ml PMA (Calbiochem, Darmstadt, Germany), 2μg/ml ionomycin (Invitrogen, Darmstadt, Germany) and 1μg/ml secretion blocker brefeldin A (Sigma-Aldrich) for 5h. Cells were fixed using a Fix/Perm buffer set (eBioscience or BD Biosciences, São Paulo, Brazil ), as described in the suppliers' manual. For intracellular staining, anti-FoxP3 APC (FJK-16s, eBioscience), anti–IL-17A APC (eBio17B7, eBioscience), anti-IL-17F FITC (9D3.1C8, BD Biosciences) and anti–IFNγ FITC (XMG1.2, BioLegend, London, UK) were used. Cytometric data were acquired on a LSRII flow cytometer (BD Biosciences) and analyzed with FlowJo software (Treestar, San Jose, CA).

*Quantification of RNA expression levels by real time PCR (qPCR)*

Cellular RNA was extracted from snap frozen recipients’ small intestine, colon, liver, or lung samples using the RNeasy Mini Kit (Qiagen, Hilden, Germany) according to the manufacturer’s protocol. Reverse transcription of 1µg of DNase I treated RNA was performed with Superscript II reverse transcriptase and random hexamers (Invitrogen). The qPCR reaction was carried out with 2xSYBR Green mastermix (Takara Holdings, Kyoto, Japan). Quantitative real-time PCR reactions were carried out on a StepOne Real-Time PCR system (Applied Biosystems, Darmstadt, Germany). The following primers were obtained from Sigma-Aldrich:

IL-21 (5’-ATGCAGCTTTTGCCTGTTTT-3’, 5’-GTTTCTTTCCTCCCCTCCTG-3’),

IL-22 (5’-GCTCAACTTCACCCTGGAAG-3’, 5’-GTCGTCACCGCTGATCTG-3’),

HPRT (5’-TCCTCCTCAGACCGCTTT-3’, 5’-CCTGGTTCATCATCGCTAATC-3’),

IL-17f (5’-TGCCATTCTGAGGGAGGTAG-3’, 5’-ACAGAAATGCCCTGGTTTTG-3’),

IL-17a (5’-TTTAACTCCCTTGGCGCAAAA-3’ 5’-CTTTCCCTCCGCATTGACAC-3’).

Melting curves of the amplicons were acquired in order to determine the specificity of the PCR reactions. Data was analyzed with StepOne software Version 2.2.2 using 0.5 cycle difference as threshold for quantification. Amounts of mRNA relative to WT T cell recipients were calculated with the comparative ΔΔC(t) method.

*Immunofluorescence staining*

Recipient mice were sacrificed and tissue (small intestine, colon, skin, lungs) was snap frozen in Tissue-Tek OCT Compound (Sakura, Torrance, USA) and cut into 6µm thick sections. The slides were fixed with -20°C cold acetone on ice for 10min. Before staining, the sections were blocked with 5% anti rat serum for 15min and washed twice with TBST. Anti-F4/80 PE (Life Technologies) was used and nuclei were stained with DAPI. Pictures were acquired with a Zeiss AxioCam (Zeiss, Oberkochen, Germany) or Olympus BX61 (Olympus, Hamburg, Germany) confocal microscope and processed with the cellSens Dimensions 1.9 software (Olympus, Hamburg, Germany).

*Cytokine bead array*

The concentrations of the inflammatory cytokines IL-6, IFN-γ, TNF and CCL2 in the sera of *Il17af^–/–^* and WT recipients at day 7, 14 and 21 after transplantation were quantified by “Mouse inflammatory cytokine cytometric bead array kit” (BD Biosciences, San Diego, USA) according to the manufacturer’s manual.

*FITC-Dextran assay*

Permeability of the intestinal barrier during the course of acute GVHD was tested by the Fluorescein isothiocyanate (FITC)-dextran assay as previously described (2). In brief, at defined time points after transplantation the mice were deprived from food and water for 4h and were subsequently gavaged with 16mg high molecular 4000mol FITC-dextran (46744, Sigma-Aldrich) solved in 300µl PBS. After additional 4h without food and water, mice were sacrificed and blood samples were collected by cardiac puncture. After 15 min of centrifugation at 4°C the serum was collected. The samples were diluted 1:1 with PBS and fluorescence was measured with a GEMINI EM multiplate reader (Molecular Devices, Sunnyvale, USA) (Ex=485 nm, Em=538 nm). A standard curve of solved FITC-Dextran was prepared to determine the exact concentration of FITC-dextran in the serum.

**References**

1. Koenecke C, Lee C-W, Thamm K, Föhse L, Schafferus M, Mittrücker H-W, et al. IFN-γ Production by Allogeneic Foxp3+ Regulatory T Cells Is Essential for Preventing Experimental Graft-versus-Host Disease. J Immunol. 2012;189:2890–6.

2. Hanash AM, Dudakov JA, Hua G, O’Connor MH, Young LF, Singer N V., et al. Interleukin-22 Protects Intestinal Stem Cells from Immune-Mediated Tissue Damage and Regulates Sensitivity to Graft versus Host Disease. Immunity. 2012 Aug;37(2):339–50.
